# Supplementary material for: Splice-Junction-Based Mapping of Alternative Isoforms in the Human Proteome
Source: Cell Rep. Author manuscript; Available in PMC 2020 Jan 15. (PMC6961840; doi:10.1016/j.celrep.2019.11.026)
Supplement: 3 [file NIHMS1546469-supplement-3.zip › DF2/PXD009737/TestisTrypsin-79-O95155-SVASFGASSLSSLGASGGASNWDSYSDHFTIETCK.pdf]

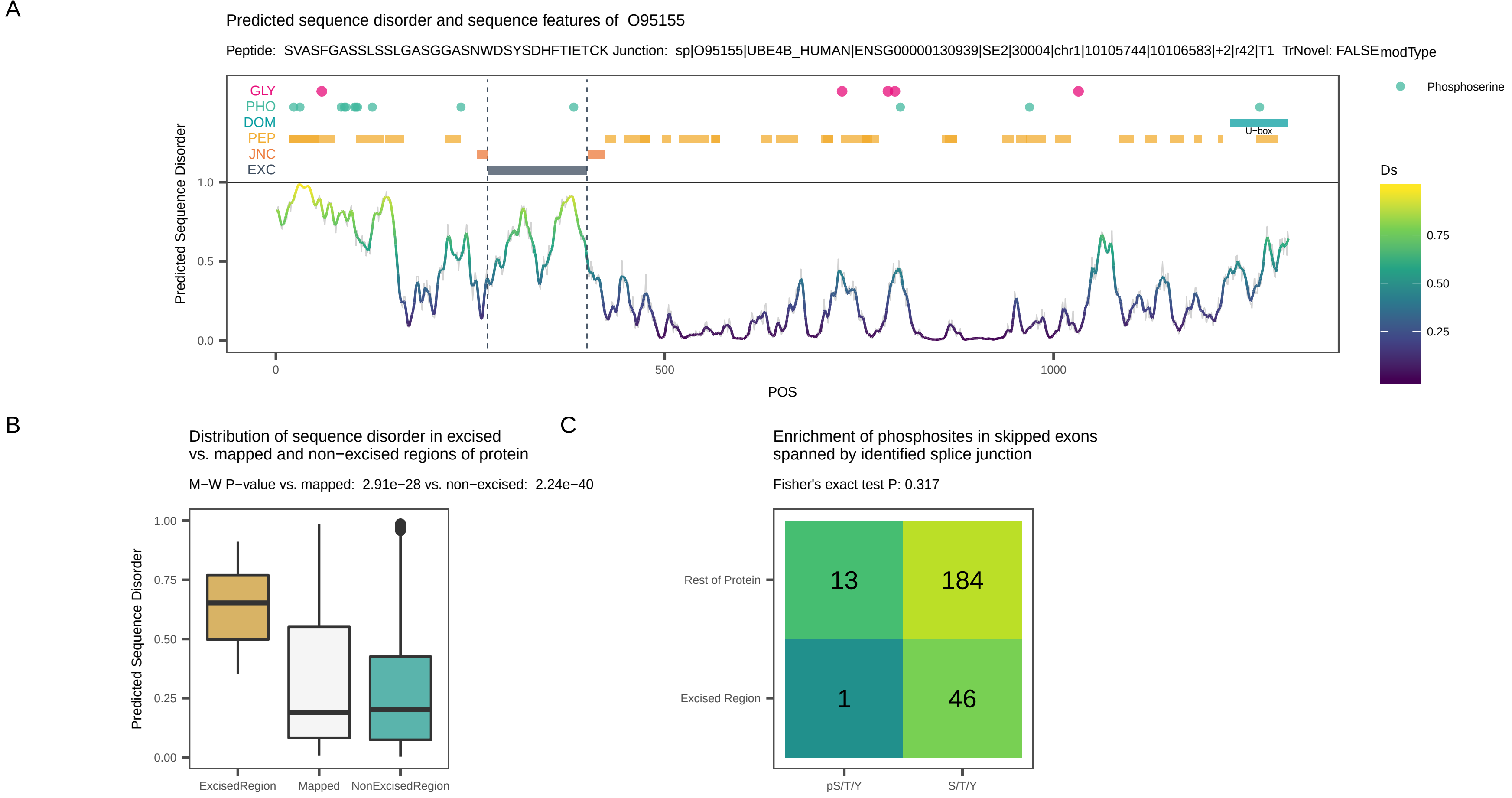
A
Predicted sequence disorder and sequence features of O95155
Peptide: SVASFGASSLSSLGASGGASNWDSYSDHFTIETCK Junction: sp|O95155|UBE4B_HUMAN|ENSG00000130939|SE2|30004|chr1|10105744|10106583|+2|r42|T1 TrNovel: FALSE
modType
Phosphoserine
GLY
PHO
DOM
PEP
JNC
EXC
U−box
Predicted Sequence Disorder
1.0
0.5
0.0
0
500
1000
POS
Ds
0.75
0.50
0.25
B
Distribution of sequence disorder in excised
vs. mapped and non−excised regions of protein
M−W P−value vs. mapped: 2.91e−28 vs. non−excised: 2.24e−40
Predicted Sequence Disorder
1.00
0.75
0.50
0.25
0.00
ExcisedRegion
Mapped
NonExcisedRegion
C
Enrichment of phosphosites in skipped exons
spanned by identified splice junction
Fisher's exact test P: 0.317
Rest of Protein
Excised Region
13
184
1
46
pS/T/Y
S/T/Y
